# The Effect of Patient Involvement in Implant Size Selection on Satisfaction With Breast Size: An Analysis of 1840 Primary Augmentations

**DOI:** 10.1093/asj/sjaf085

**Published:** 2025-05-16

**Authors:** Jerzy Kolasiński, Małgorzata Kolenda, Szymon Kołacz, Anna Kasielska-Trojan

## Abstract

**Background:**

Aesthetic breast surgery is a specific field in which the aim is not to achieve any universally defined standard but to meet the patient's expectations.

**Objectives:**

The aim of this study was to determine the proportion of women who were dissatisfied with their breast size following primary breast augmentation and opted to undergo revision surgery. The study presents and verifies an algorithm for breast implant selection which involves the patient in the decision-making process.

**Methods:**

A retrospective analysis was performed of medical charts covering the period from January 2012 to December 2022 from 1 private center. The final analysis included 1840 patients. All had implants chosen according to the Patient Decision-Making Process on Implant Size Selection (PIS) algorithm.

**Results:**

Of the studied cases, 18 opted to undergo implant exchange due to dissatisfaction with breast size (0.98%). This group differed significantly from the controls (n = 1822) in the following aspects: lower BMI during primary procedure, lower mean implant volume, and shorter follow-up. In all but 2 women, mean BMI increased by 1 unit before revisional surgery (from 19.7 to 20.6 kg/m^2^).

**Conclusions:**

Dissatisfaction with breast implant size is a rare cause of revision in breast augmentation surgery when the patient is involved in the final decision on the volume of the implants. The PIS algorithm yielded a rate of dissatisfaction with breast size of less than 1% in long-term follow-up.

**Level of Evidence: 4 (Therapeutic):**

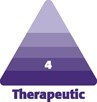

Breast augmentation, one of the most popular cosmetic surgical procedures globally, aims at improving the physical appearance, self-esteem, and quality of life of the patient. According to statistics from the American Society of Plastic Surgeons, 304,181 breast augmentations using implants were performed in the United States in 2023.^1^

Although complications may occur in the theater, a major concern for breast surgeons is patient dissatisfaction, which can often result in psychological distress, decreased quality of life, or even the need for revision surgery.^2,3^ The need for revision most often stems from technical complications, such as capsular contracture, implant displacement, and/or rupture or seroma; however, it can also be driven by unmet patient expectations, which are often shaped by social environment pressures and media influence.^4-6^ Moreover, pre-existing psychological conditions, such as neuroticism or body dysmorphic disorder, can also influence the perception of the augmented breast and increase the chance that revision surgery will be requested.^3,7^ A recent analysis by Danilla et al found greater dissatisfaction to be associated with increased BMI, the passage of time since the operation, and misinformation; the authors highlighted the need for patient education and expectation management to enhance postsurgical satisfaction.^8^ However, a study by Brown did not identify BMI as a predictor of early dissatisfaction with breast implant size based on regression analysis. Brown concluded that it is essential to encourage clear communication about the potential risks and results of treatment to ensure patient satisfaction and reduce the likelihood of postoperative regret.^4^ Surgeons should regularly obtain feedback regarding patient satisfaction, as this can also enhance provision of informed consent.^4,5^

Apart from patient-related characteristics, key considerations in the preoperative procedure are the way in which breast implants are chosen and patients are educated. The choice of breast implant may be based on 2 distinct approaches or a combination thereof, namely, reliance on a measurement system such as Tebbetts and Adams's original “high five,” or on the aesthetic requirements of the patient.^9^ Most often surgeons use a combined approach, in which they apply a combination of measurements and mathematical rules, as well as listening to the patient's preferences.^10,11^ Both approaches have similar reoperation rates for size-related issues, ie, less than 5%.^12^

Although previous studies have identified several anthropometric (eg, BMI), demographic, and psychological features as risk factors influencing dissatisfaction with breast augmentation, this study focuses on dissatisfaction with breast size following primary breast augmentation as a cause of revision. No such long-term analysis has previously been performed. The study also presents and verifies a new algorithm for breast implant selection that involves the patient in the decision-making process.

## METHODS

### Study Design

The study was designed as a retrospective analysis of medical charts from a single private plastic surgery center. The senior author developed the key study variables and performed the analysis: the study included as patients those who underwent primary breast augmentation in the center between January 2012 and December 2022. The following inclusion criteria were applied: women who underwent primary breast augmentation, regardless of type of implant and cosmetic diagnosis (eg, tubular deformity) in the clinic, who attended the follow-up, and whose implant selection process was based on the Patient's Decision-Making Process on Implant Size Selection (PIS) algorithm; this algorithm was designed by the senior author and has been applied in the plastic surgery center for 20 years.

Patients who underwent reconstruction or symmetrization after mastectomy due to breast cancer were excluded. The analyzed data included patient demographic variables, such as age, education, number of children, weight, and height. The data also included, where relevant, a history of breast implant revision, looking at the reason for implant removal or exchange (focusing on the group of women who exchanged implants due to dissatisfaction with their size). Information about the pocket of the implant (subfascial vs dual plane) was also recorded.

### The PIS algorithm

**Figure 1.**
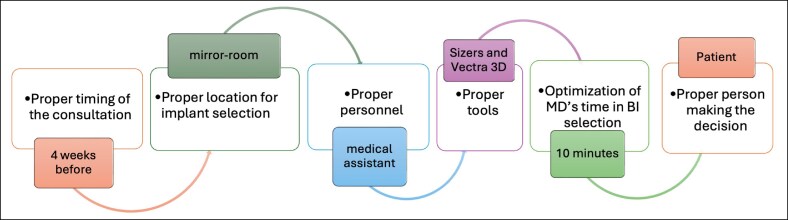
A scheme presenting the Patient Decision-Making Process on Implant Size Selection (PIS) algorithm.

**Figure 2.**
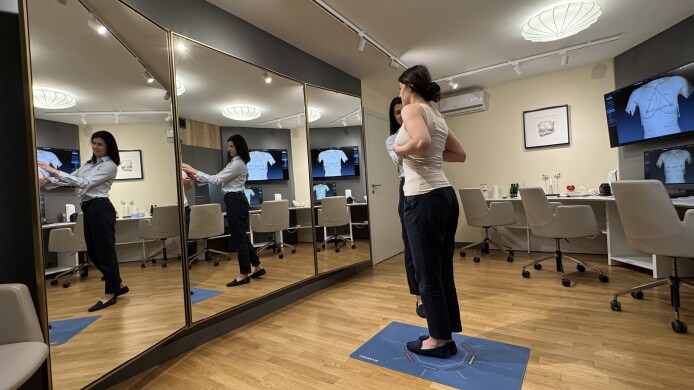
A room dedicated exclusively to selecting breast implants is equipped with a system of large mirrors.

**Figure 3.**
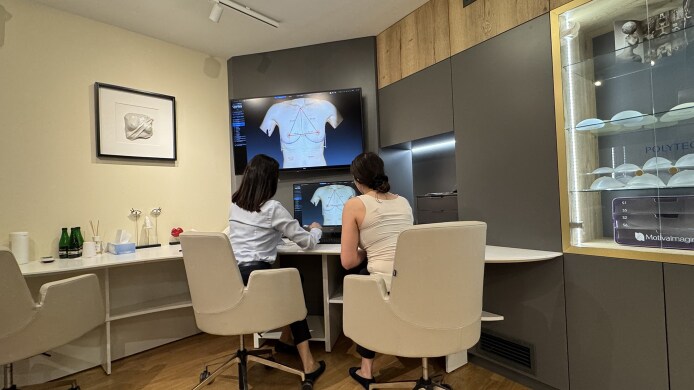
The Vectra 3D (Canfield Scientific, Parsippany, NJ) system provides a comprehensive 3-dimensional analysis of the patient's chest and breast.

The main aim of the implant selection process is to include the patient in the final decision regarding the size of breast implants; in this process, the surgeon's role is to provide an overview of the possible effects and a range of possible shapes and volumes. The selection of breast implants through the PIS algorithm is based on 6 pillars (Figure 1):

1. Proper timing. The appropriate time for selecting implants is believed to be 1 month before surgery. This period is sufficiently distant from the operation date to prevent the patient from experiencing stress related to the upcoming procedure, while also being close enough to the surgery that the patient is decisive.
2. Proper location. The consultation takes place in a dedicated space, ensuring a comfortable atmosphere. The room is equipped with a system of large mirrors, allowing the patient to view her figure from various angles. A key element of the space is a large monitor connected to a Vectra 3D (Canfield Scientific, Parsippany, NJ) imaging and visualization system (Figure 2).
3. Proper personnel. Breast implant selection is conducted with the support of a medical assistant. This is a person experienced in dealing with patients (ie, at least 3 years of working as a patient assistant at the reception), who completed training in the clinic and is familiar with chest anatomy, types of implants, and the rules for implant selection according to chest morphology, and has at least basic psychological education. This individual demonstrates high empathy in patient interactions and is proficient in using sizers and the Vectra 3D system. The estimated time commitment for the assistant is 30 to 60 minutes. The document reporting the visit is always included in patient's medical records.
4. Proper tools. The selection process utilizes a set of sizers in combination with appropriate bras and T-shirts. Another key element is the Vectra 3D system, which enables a comprehensive 3-dimensional (3D) analysis of the patient's chest and breasts, provides linear and volumetric measurements, and simulates the expected results with specific breast implants (Figure 3).
5. Optimization of the physician's time. In the PIS system, the physician's role is limited to the final verification of the selection of implant volume by the patient. Based on the patient's preferences and the measurements of the chest and breasts, obtained by the Vectra 3D analysis, the physician determines implant parameters such as width, height, and projection. Whenever possible, the physician respects the patient's volume preference. Additionally, the physician is responsible for answering all patient inquiries. Typically, the physician's involvement in the implant selection process takes 5 to 10 minutes.
6. Proper person (patient) making the decision. This structured implant selection process ensures that the final decision regarding breast implants is made by the patient herself. The medical assistant and physician provide full advisory support in this regard. The use of sizers and the Vectra 3D system allows for proper patient education about the initial anatomical state of the chest and breasts and provides simulations to assist the patient with her decision.

### Breast Implant Selection Process

Only patients who have already decided to undergo breast augmentation, ie, those who have already attended the initial consultation, qualify for the implant selection consultation. As a rule, this consultation is intended to answer the question of which implants to choose, rather than whether to undergo breast augmentation. Because all essential information regarding preoperative preparation, the procedure itself, recovery, and the risks of possible complications was previously discussed during the initial consultation, the selection consultation is therefore not intended to encourage patients to undergo the procedure but is strictly dedicated to selecting the appropriate implant size and parameters.

**Figure 4.**
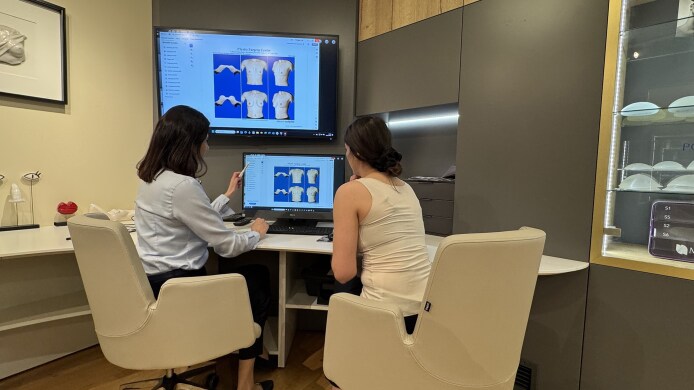
The Vectra 3D (Canfield Scientific, Parsippany, NJ) system displays a simulation of the expected results, which helps the patient with the final choice of specific breast implants.

At the beginning of the consultation, the medical consultant takes a photograph of the patient using the Vectra 3D camera, and analyzes all linear and volumetric anatomical parameters, and asymmetries, of her chest and breasts with her. Next, using sizers, the patient selects the implant volume. Viewing her full figure in large mirrors allows her to adjust the implant size to her body proportions. Once the implant volume is chosen, the physician joins the process to verify the dimensions provided by the Vectra 3D system by performing a traditional assessment; the physician also evaluates skin elasticity, measures the nipple–inframammary fold distance, and recommends a specific implant based on the patient's volume preference. The physician is also available to answer any questions the patient may have. Subsequently, the medical assistant enters the selected implants into the Vectra 3D system, displaying a simulation of the expected results on a large screen. The breast implant selection process concludes with the patient signing a declaration stating that she has made the final choice of specific breast implants (Figure 4). The patient is provided with the images of the chosen result or results (if hesitating between options) and, if not sure about the choice, is supposed to make a final decision within days after the visit.

After the surgery and perioperative period, all women are asked to attend follow-up visits every year for ultrasound examination. In the case of dissatisfaction regarding size or the effect, even years after the procedure, women are informed about the preferential financial terms for revisional surgery, which encourage them to perform all revisions in the clinic. This procedure provides a late follow-up rate of more than 95%.

### Statistical analysis

The normality of the data distribution for each parameter was analyzed with the Shapiro-Wilk test. The patents were divided into a study group comprising patents who opted to change their breast implants due to dissatisfaction with the size (n = 18), and a control group, comprising the remainder (n = 1822). The 2 groups were compared with regard to the collected demographic, clinical, and implant-related patient data.

The choice of test depended on the normality of distribution, homogeneity of variance, and distribution of differences. Hence, a *t*-test was used to compare groups with regard to age, the Cochran-Cox test was used for BMI and time of follow-up, the Mann-Whitney test was used for mean implant volume = (right + left implant volumes)/2, and the chi-squared test was used for number of children, residency, and type of implant (round vs anatomical). The Wilcoxon test was applied to compare the BMI of the patients between the primary and secondary procedures.

## RESULTS

### Group Demographics

After applying the inclusion and exclusion criteria, 1840 patients were included in the analysis. All attended the late follow-up visit 2 years after the procedure and had complete medical charts. Their mean [standard deviation] age was 32.9 [6.4] years (range, 18-53 years). All women were from one country (Poland) and of a single ethnicity (Caucasian). Their BMIs ranged from 15 to 29 kg/m^2^ (mean, 21.1 kg/m^2^). The mean follow-up time after surgery was 7.2 years (range, 3-10 years). The mean number of children was 1.3 per patient (range, 0-3). The education level was as follows: higher, n = 1067 (58%); secondary, n = 644 (35%); and lower or no information, n = 129 (7%). The place of residence was large cities (>100,000 citizens), n = 432 (23.5%); medium cities (30-100,000), n = 169 (9.2%); small cities (<30,000), n = 127 (6.9%); and villages (<10,000), n = 1112 (60.4%).

### Breast Surgery Details for the Whole Group

The following implant shapes were selected: round (n = 699; 37.99% of all first procedures), form-stable anatomical implant (n = 651; 35.38%), and ergonomic with a round base (n = 490; 26.63%). The mean implant volume was 337 [60] cc. The implants used were Mentor (n = 699; 37.99% of all first procedures; Mentor Worldwide LLC, Santa Barbara, CA), Polytech (n = 460; 25%; Polytech Health & Aesthetics, Dieburg, Germany) Motiva (n = 490; 26.63%; Establishment Labs Holdings Inc., Alajuela, Costa Rica), and Allergan (n = 191; 10.38%; Allergan Aesthetics, an AbbVie Company, Irvine, CA). Regarding the procedure, the implants were placed using the dual-plane technique in 1519 patients (82.55%) and the subfascial technique in 321 patients (17.45%); all procedures used an incision approach through the inframammary fold. The patients were operated on by 3 plastic surgeons in the following proportions: senior surgeon, n = 1120 (60.87% of all first procedures); M.K., n = 563 (30.60%); and S.K., n = 157 (8.53%).

### Group of Interest: Implant Change Due to Dissatisfaction With Implant Size

Eighteen patients (Table 1) opted to undergo an implant exchange due to dissatisfaction with breast size (0.98% of all patients). All patients selected larger implants for the revision, increasing from a mean volume of 293 cc to 475 cc. Within the group, the unsatisfactory implants were anatomical (n = 7) or round (n = 11) implants; these were exchanged for round (n = 11), Ergonomix (n = 6; Motiva, Establishment Labs Holdings Inc.), or anatomical (n = 1) implants. The choice of original implant type was not significantly different from the controls (χ^2^ = 0.098, *P* = .95). The mean time from primary augmentation to the secondary procedure was 3.64 years (range, 0.5-6 years). All patients had implants placed in dual-plane pockets via inframammary fold incision.

**Table 1.** Characteristics of 18 Patients Who Decided on Breast Implant Revision Due to Dissatisfaction With Breast Size

	Age (years)	Number of children presurgery	Residency^a^	Weight (kg)	Height (cm)	BMI (kg/m^2^)	Mean implant volume (cc)	Implant type^b^	Time between primary surgery and revision (years)	BMI during revision (kg/m^2^)
1	28	1	4	53	168	18.8	240.0	1	1	18.8
2	44	2	3	54	167	19.4	325.0	2	3	20.8
3	25	0	4	61	170	21.1	350.0	2	6	21.8
4	36	1	2	51	164	19.0	350.0	2	2	19.3
5	31	0	1	53	170	18.3	325.0	2	5	20.7
6	29	1	2	47	160	18.4	325.0	2	9	20.7
7	32	2	2	53	165	19.5	275.0	2	2	20.2
8	37	1	4	53	173	17.7	237.0	2	1	18.1
9	28	1	3	52	164	19.3	375.0	2	6	17.9
10	31	1	1	54.7	169	18.9	300.0	2	0.5	19.2
11	36	2	4	56	170	19.4	210.0	1	4	19.9
12	36	1	2	62	172	20.9	225.0	1	4	23.3
13	38	3	3	57	170	19.7	350.0	1	2	20.4
14	43	0	2	64.5	171	21.9	265.0	1	3	22.6
15	31	0	3	46	164	17.1	237.5	2	5	17.4
16	35	2	4	71	185	20.7	300.0	2	4	21.6
17	32	0	3	63	162	24.0	297.5	1	4	26.3
18	34	1	2	54	164	20.8	295.0	1	4	21.8

^a^1, big city (>100,000 citizens); 2, medium city (30-100,000); 3, small (<30,000); 4, village (<10,000).

^b^1, anatomical; 2, round; 3, Ergonomix.

The group of interest differed significantly from the controls with regard to the following aspects: lower BMI during primary procedure, lower mean implant volume, and shorter follow-up (Table 2). However, they did not differ regarding age during the procedure, residency, or number of children (χ^2^ = 4.437, *P* = .22).

**Table 2.** Characteristics of Women Who Elected for Breast Revision Due to Dissatisfaction With Breast Size (n = 18) Versus Those Satisfied With Breast Size

					Test	Value	CI	*P*	Cohen's *d*
	n	Mean [SD]	Median	(25%-75%)					
Age (years)					t	0.5	(−2.21 to 3.72)	.62	0.131
GOI	18	33.67 [5.03]	33	(31-36)					No effect
Controls	1822	32.91 [6.39]	32.92	(28.6-37.3)					
BMI (primary procedure, kg/m^2^)					CC	−3.52	(−2.48 to −0.27)	.002	−0.67
GOI	18	19.72 [1.64]	19.4	(18.8-20.8)					Medium
Controls	1822	21.09 [2.39]	21.12	(19.4-22.7)					
Mean implant volume^a^ (cc)					MW	−3.12		.002	−0.81
GOI	18	293.47 [49.26]	298.75	(240-325)					Large
Controls	1822	337.62 [59.32]	336.25	(300-380)					
Length of follow-up (years)					CC	−7.13	(−4.07 to −3.09)	<.0001	−2.14
GOI	18	3.64 [2.13]	4	(2-5)					Large
Controls	1822	7.22 [1.04]	7.21	(6.57-7.98)					

GOI, group of interest (women who had breast implant revision due to dissatisfaction with breast size); CC, Cochran-Cox test; MW, Mann-Whitney test; SD, standard deviation. ^a^Mean implant volume = (right implant cc + left implant cc)/2.

In all but 2 women from the revision group, BMI increased before revisional surgery: the mean BMI increased from 19.7 to 20.6 kg/m^2^ among these patients. Among the other 2, no change was noted in 1 patient, and a decrease of about 1.4 units in the other (Table 3).

**Table 3.** Difference in Mean BMI Between Primary and Secondary Augmentation

					Value	CI	*P*	Cohen's *d*
	N	Mean [SD]	Median	(25%-75%)				
BMI (primary procedure, kg/m^2^)	18	19.72 [1.64]	19.4	(18.8-20.8)	12.5	(35-∞)	.0024	−0.9 Large effect
BMI (secondary procedure, kg/m^2^)	18	20.6 [2.16]	20.55	(19.2-21.8)				
Difference BMI (kg/m^2^)	18	0.88 [0.98]	0.7	(0.32-1.3)				

SD, standard deviation.

## DISCUSSION

This paper presents a scheme for breast implant selection, the PIS algorithm, in which the final decision about the volume of the implant is influenced by the preferences of the patient. The scheme also employs external sizers and various measurements and simulations to better understand patient expectations. The PIS algorithm was generally successful, with fewer than 1% of patients opting for reoperation due to dissatisfaction with breast size.

Breast aesthetic surgery is a specific field in which the aim is not to achieve any universally defined standard but to meet the patient's expectations. These are often difficult to define, and most women ask for estimation of a postsurgical outcome. It is possible to estimate the shape and volume of the desired augmented breast using a variety of presurgical tools, such as measuring systems provided by breast implant manufacturers, dedicated software, and/or breast implant sizers, as well as simple placement of surgical swabs into a bra.^9,13,14^ A particularly common approach to implant selection is based on objective measurements, emphasizing the base diameter of the breast as one of the critical variables. This approach is often suggested by device manufacturers and included in the software used for implant selection.

Nevertheless, many patents elect to return to the operating room after breast augmentation to replace the implant with one of a different size.^4,12,15-20^ This may be related to the effect observed by Hammond, namely, that the acceptable widths of implant bases may not vary considerably but result in more significant variability in volumes. Furthermore, the final size can have a significant effect on patient satisfaction after breast augmentation.^21^

Hence, Hammond et al recommend that the primary variable in implant selection is to choose the volume of the implant together with the patient and then adjust other device specifications.^22^ The concept of involving the patient in deciding on the implant seems to be increasingly common. Indeed, it has been suggested that active patient involvement in the process shifts some responsibility for the final effect to the patient, both morally and legally.^4^

However, this raises the question of which variable should be discussed with the patient, and whether the volume is suitable. This variable is well understood by patients, but often they compare volumes between different postoperative results without considering their chest characteristics, their weight and height, and their preoperative breast size. To support the decision, many commercial systems and applications based on 3D visualizations are available. These systems can be used to present a patient with images of the predicted result with different volumes and types of implants.^23-25^ Their effectiveness in educating patients and predicting the size after surgery may depend on many variables and is not universal.^26-28^

A study of size-related satisfaction of augmented patients by Hammond et al examined the value of volume as a primary variable in breast implant selection.^22^ The findings indicate that volume was an effective guide when discussed together with other measured parameters. This resulted in high levels of patient satisfaction, with no reoperations for size change in the examined group of patients (n = 40). Hammond et al concluded that the most effective methods of implant size selection include the use of 3D imaging (ie, Crisalix, Crisalix SA, Lausanne, Switzerland) and external sizers, which both enhance effective communication with the patient.^22^

There are numerous studies on the role of 3D simulations and the use of specific devices and software in enhancing preoperative planning of breast augmentation by offering a preview of possible results. The Vectra 3D system has been proven to be accurate in predicting the volumetric outcomes of breast procedures, achieving >90% accuracy. Moreover, simulating certain proportions and shapes improves communication between patients and surgeons, leading to higher satisfaction rates with the surgical effects.^27,29,30^ This influenced the trend that most patients prefer clinics which offered 3D technology and claim that the simulation helped them in choosing the implant.^31^ Montemurro et al critically analyzed their experience with breast 3D simulations and placed their role in the process of selecting appropriate implants as a final complement, after choosing implants according to anthropometric features, trying the chosen sizers, and then presenting a simulation with the chosen implant to help the patient visualize the result. They strongly suggest not showing simulations of implants that are not suitable for the patient based on anthropometric features.^32^ In this aspect another potential role of such simulations should be highlighted. They enable patients to conceptualize the effect not only in the volumetric aspect but also in the aspect of breast shape and proportions. As pointed by Montemurro et al, proper use of Vectra simulations provides realistic previews of the accurately chosen implants, which by enhancing communication with the patient improves satisfaction rate.

The idea of patient inclusion in the decision-making process has been part of the present authors’ practice since 2010, and during the first year a system was designed incorporate this. The analysis included patients whose implant selection was based on the PIS schema described above; after 1 year of implementation in the clinic, this was refined into its final form. Similarly to the approach presented by Hammond et al,^22^ our system is also based on the patients’ selection of implant volume, which is in turn based on their preferred body image and proportions observed in mirrors. However, the patient is not involved in the initial selection of possible implants: their inclusion in the decision process starts when different visualizations, including the preferred volumes, are presented. This enables the patient to focus their search within a given range of options based on images presenting the possible results with different implants. This approach entails using simulations not to predict the effect but to better understand patient's expectations. The patient should then make her decision on the implants according to the chosen effect (ie, the implant volume). Some patients decide to provide their decision on volume within 1 week after consultation. If they are unable to do this and instead “rely on the surgeon's opinion,” they are recommended to go through the process again. Here, we highlight the role of tools such as sizers and the Vectra 3D system for informing patients about different aspects of their anatomy and about the possible results of the surgery, which is crucial for proper communication and patient education, and consequently for patient satisfaction.

The present analysis focuses on revision as an objective measure of dissatisfaction with breast size. This contrasts with previous papers, which present data about dissatisfaction from breast size based on the subjective opinion of the patient. Such an approach may overestimate the true rate of dissatisfaction because some women who declare they would prefer larger implants subsequently choose not to undergo revision. Therefore, evaluations of different selection systems, apart from complication rates, should include at least 10-year revisions due to dissatisfaction with size. Such data could allow evaluations of the clinical value of a particular approach and encourage comparisons between them. Furthermore, it would be beneficial to compare different algorithms utilizing different tools for implant selection (Vectra 3D, sizers, measurements, software for result simulation) in the aspect of patients’ satisfaction and the rate of revisions. A systematic review of this aspect should be designed and performed.

Regarding the factors associated with implant revision due to dissatisfaction with breast size, our findings indicate that this group tended to have a lower BMI before primary augmentation compared with the women who were satisfied with the result. Their BMI also appeared to be significantly higher before revision surgery. This highlights the role of stable body mass in maintaining the initial effect of breast surgery, be it augmentation, reduction, and/or lifting. Due to inconsistent data on the impact of BMI on breast implant revision rate,^4,8^ it is possible that it is not the BMI itself, but rather its change, that may be related to subsequent dissatisfaction with breast size following augmentation. Other demographic factors seemed not to affect the decision for revision related to implant sizes.

The presented analysis has some limitations. It includes only women who met the study criteria and attented the free follow-up meeting annually for at least 2 consecutive years after primary augmentation, and who decided to undergo breast revision in the same clinic due to dissatisfaction with breast size. No attempt was made to retrospectively contact patients and ask about revisions performed elsewhere. However, the clinic where the study was performed offers preferential financing for revisions carried out there, and it is highly likely that patients would choose to return for later procedures whenever possible. Some rare cases of women who were unavailable for such follow-up due to, for example, moving abroad were estimated to amount to less than 5%.^33^ Additionally, because we included patients of only a single ethnic origin (Caucasians), the accuracy of our algorithm for implant selection may not apply to other ethnicities due to different aesthetic expectations. To provide such data, the algorithm should be verified in a more diverse population. Also, other factors possibly associated with revisions due to dissatisfaction with breast size, eg, psychosocial factors, should be considered in future studies.

## CONCLUSIONS

Although dissatisfaction with breast implant size is a cause for revision, such cases are relatively rare when the patient is involved in the final decision on the volume of their breast implant. The PIS algorithm presented here prioritizes the role of the patent in selecting implant size by involving sizer use and Vectra 3D simulations to understand patient's expectations and requires a final decision to be made by a patient. The presented system, based on 6 pillars, resulted in over 99% of patients being satisfied with their decision in a long-term follow-up. This is the highest rate reported so far in the literature.
